# Octanol-assisted liposome assembly on chip

**DOI:** 10.1038/ncomms10447

**Published:** 2016-01-22

**Authors:** Siddharth Deshpande, Yaron Caspi, Anna E. C. Meijering, Cees Dekker

**Affiliations:** 1 Department of Bionanoscience, Kavli Institute of Nanoscience, Delft University of Technology, Lorentzweg 1, 2628 CJ Delft, The Netherlands

## Abstract

Liposomes are versatile supramolecular assemblies widely used in basic and applied sciences. Here we present a novel microfluidics-based method, octanol-assisted liposome assembly (OLA), to form monodisperse, cell-sized (5–20 μm), unilamellar liposomes with excellent encapsulation efficiency. Akin to bubble blowing, an inner aqueous phase and a surrounding lipid-carrying 1-octanol phase is pinched off by outer fluid streams. Such hydrodynamic flow focusing results in double-emulsion droplets that spontaneously develop a side-connected 1-octanol pocket. Owing to interfacial energy minimization, the pocket splits off to yield fully assembled solvent-free liposomes within minutes. This solves the long-standing fundamental problem of prolonged presence of residual oil in the liposome bilayer. We demonstrate the unilamellarity of liposomes with functional α-haemolysin protein pores in the membrane and validate the biocompatibility by inner leaflet localization of bacterial divisome proteins (FtsZ and ZipA). OLA offers a versatile platform for future analytical tools, delivery systems, nanoreactors and synthetic cells.

## Abstract (web summary)

A broad application of liposomes calls for high throughout techniques to produce them in a controlled and fast manner. Here, Deshpande *et al*. show a microfluidic approach using alcohol-based lipid-carrying material to generate monodisperse and unilamellar liposomes within a just few minutes.

## Graphical abstract

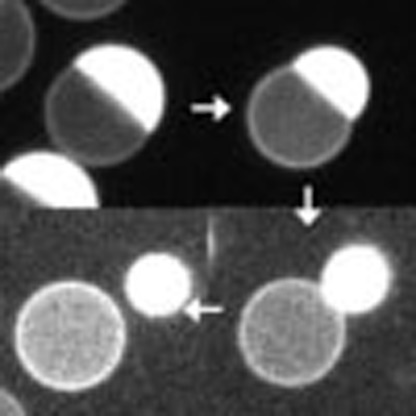

Liposomes are microscopic aqueous compartments confined by a lipid bilayer that separates them from the surrounding aqueous environment. Over the years, liposomes have become an important and versatile tool in science, medicine and industry. They serve as an essential tool in membrane science^1^, for example, to study membrane transport processes^2^ as well as fission and fusion of vesicles^3^,^4^. They can act as nanocontainers to reconstitute cytoskeletal polymers^5^,^6^ or as bioreactors with a diffusive transport across the membranes^7^. On the application side, liposomes have emerged as an important new carrier for efficient drug and gene delivery^8^, and they are a useful analytical tool, for example, in immunoassays, biosensors and liposome-nanotube networks^9^. Finally, they mimic a minimalistic model system of living cells^10^, and thus lie at the very heart of creating synthetic cells, providing an essential scaffold for the bottom-up approach to build cell-like objects from individual components. Indeed, in recent years they have become a model system for understanding bacterial cell division, with the final goal of constricting and dividing liposomes using bacterial divisome proteins^11^,^12^,^13^.

With such a wide array of liposome-related disciplines, it is extremely useful to develop methods to produce liposomes in a controlled, robust and simple way. Ideally, one would favour unilamellarity, good encapsulation efficiency, a wide range of sizes, monodispersity, a high production rate and adept post-production control when developing the best method. Traditionally, various methods have been developed for the bulk production of liposomes, such as hydration^14^, extrusion^15^ and electroformation^16^. Although relatively simple to implement, they suffer from major drawbacks such as a high polydispersity and low encapsulation efficiency^17^. These processes are also discontinuous, that is, the lipid source always needs replenishment.

Microfluidics offers a highly controlled and reproducible environment to produce liposomes on chip. In recent years, a number of microfluidic methods have been introduced to generate liposomes. An interesting approach involves forming water-in-organic solvent-in-water double-emulsion droplets, followed by slowly extracting the organic solvent to let the dissolved lipids form a bilayer^18^. Ultrathin-shelled double-emulsion droplets can be formed using such a technique, albeit carrying a pocket of extra lipids and residual solvent^19^. The same principle was adapted to a polydimethylsiloxane (PDMS)-based microfluidic device, with oleic acid as the lipid-carrying phase^20^, involving, however, a very long incubation time (>15 h) that is required to extract the oleic acid to form a liposome. This renders the method impractical for biomaterial-based applications where molecular degradation and energy consumption by encapsulated enzymes are important. Other techniques such as pulsed jetting^21^,^22^ and various phase-transfer methods^23^,^24^,^25^,^26^ have been developed but suffer from low yields, discontinuous operation and again, remnants of oil in the membrane. Thus, while the existing techniques are very useful in specific applications, a common major disadvantage is the use of oil as the lipid-carrying solvent and the inability to remove the associated oil remnants from the liposome bilayer in a quick and efficient manner.

In this paper, we present a new and robust microfluidic technique, octanol-assisted liposome assembly (OLA), to form unilamellar, monodisperse, cell-sized liposomes with an efficient, autonomous and fast solvent-extraction mechanism. Using a double-emulsion-droplet-based approach, we replace the conventional oil-based or alkane-based lipid-carrying phases with an alcohol-based one. Double-emulsion droplets formed in OLA quickly develop into an intermediate complex of an aqueous lumen encapsulated by a lipid bilayer interface and a 1-octanol pocket connected to it. In stark contrast to the very long solvent-extraction time needed in other double-emulsion-droplet-based methods, the 1-octanol pocket is found to spontaneously split off from the liposome within just a few minutes, leaving behind a fully maturated liposome. The efficient, non-leaky pinching-off process ensures excellent encapsulation efficiency. We prove the unilamellarity of liposomes by inserting functional α-haemolysin pores. Furthermore, we demonstrate the localization of encapsulated bacterial divisome proteins (FtsZ and ZipA) to the membrane, which additionally serves to show the biocompatibility of the vesicles. The liposomes are formed in biologically relevant sizes (5–20 μm) and display high monodispersity (coefficient of variation 4–11%). Moreover, OLA is robust to physiological salt concentrations and variations in the bilayer composition, rendering it a very versatile high-throughput technique for forming liposomes on chip.

## 

### Octanol-assisted liposome assembly

We developed a microfluidic design for OLA consisting of a six-way PDMS junction (Fig. 1a): One inner aqueous phase (IA) channel, two lipid-carrying organic phase (LO) channels, two outer aqueous phase (OA) channels and a downstream channel. Structures were defined in PDMS after e-beam lithography of a silicon template (see Methods). A crucial step was to control the hydrophobicity/hydrophilicity of the channels. To prevent the adsorption of liposomes on the surface, the post-junction area was rendered hydrophilic by coating it with polyvinyl alcohol (PVA; Supplementary Fig. 1; Methods). Our initial idea was to use an LO phase that readily dissolves lipids, has a high double-emulsion-droplet-forming potential and is also partially miscible in water, to quicken the solvent-extraction process after double-emulsion droplet formation (as in the scheme in Fig. 1a).

With our PVA-coated microfluidics devices, we started to form double-emulsion droplets with the widely used oleic acid as the LO phase. Using pressure-driven flow pumps and benefitting from a single six-way junction, the IA stream and the surrounding LO streams were hydrodynamically focused and subsequently pinched off by the two OA streams (Fig. 1a,b). Thus, akin to the process of bubble blowing, stable and monodisperse double-emulsion droplets formed in a single step. The inset in Fig. 1b shows, however, a prominent and stable oleic acid pocket that is formed after a few minutes. The full extraction of oleic acid from these double-emulsion droplets using 10 v% ethanol was highly time-consuming, requiring more than 10 h, as documented previously^20^. As alcohols are good lipid solvents and also partially miscible in water (the miscibility inversely proportional to the number of carbon atoms present), we set out to systematically check the suitability of different alcohols as the LO phase (see Supplementary Table 1 for more details). A mixture of 10 v% oleic acid and 90 v% 2-propanol resulted in smaller oil pockets, since the miscible fraction (2-propanol) rapidly went into the aqueous phase (Fig. 1c). However, residual oleic acid remained whose extraction was still equally time consuming. Use of 2-butanol (without any oil) did give rise to double-emulsion droplets, but in a very uncontrolled way, yielding thick shells with lipid aggregates (Fig. 1d). A marked improvement was seen on using 1-octanol as the LO phase as compared with all the other LO phases that were tested. Stable and monodisperse double-emulsion droplets were formed, and by carefully choosing the flow velocities of the three phases, ultrathin-shelled double-emulsion droplets could be formed resulting in very small alcohol pockets (Fig. 1e). Although these smaller pockets present a clear advantage, formation of such ultrathin-shelled double-emulsion droplets was susceptible to even slight perturbations in the flow rates of the different phases and hence required a very precise control. However, one key observation concerning these 1-octanol-induced double-emulsion droplets—the dynamics of the alcohol droplet—helped us to uncover the true potential of using 1-octanol as the LO phase.

By increasing the amount of the LO phase to form the double-emulsion droplet, we found a novel way of producing well-defined liposomes. As can be seen in step I of Fig. 2, stable and monodisperse double-emulsion droplets were formed in a single step, with typical production rates between 25 and 75 Hz (Supplementary Movie 1). Once formed, the LO phase distributed asymmetrically across its surface, forming a distinct crescent-shaped volume at one side (Fig. 2, step II). As a result, within a few seconds after its formation, the double-emulsion droplet developed into an intermediate complex containing two distinct phases: a prominent pocket of 1-octanol containing excess lipids; and an inner aqueous lumen surrounded by a lipid bilayer (Supplementary Movie 2). Interestingly, unlike the static oleic acid pocket, the 1-octanol pocket continued to grow and protrude outwards with time. The interfacial area between the encapsulated IA phase and the pocket continuously reduced in size and within a few minutes, the 1-octanol droplet completely budded off from the fully assembled liposome (Fig. 2, step III). This spontaneous budding-off process is highly efficient and occurs for virtually every double-emulsion droplet. Just within 5 min, 85% of double-emulsion droplets separated into liposomes and 1-octanol droplets (*n*=444). In 8 min, the efficiency further increased to 95% (*n*=339). Also, only a small percentage of the formed liposomes (4%) had some minute but unwanted lipid deposits attached to them (*n*=671). Figure 2c exemplifies the last stages of such a separation (Supplementary Movie 3). This separation of 1-octanol pocket from the liposome can be understood as resulting from a combination of energy minimization and some shear stress induced by the surrounding fluid streams. The interfacial tension between 1-octanol and water is 8.5 mN m^−1^ (ref. 27) while the membrane tension of a liposome is very low, in the order of few μN m^−1^ (ref. 28). Furthermore, the presence of short 1-octanol molecules (8 carbon atoms) amongst longer lipid molecules (18 carbon atoms in this case) is energetically unfavourable as it forms defects in the ordered structure of lipids and can disrupt the hydrophobic interactions between their tails; it has been shown that the lipid bilayer tension increases considerably in presence of 1-octanol^29^. These factors together induce the energetically favourable self-assembly of a lipid bilayer along the double-emulsion droplet interface, continuously pushing 1-octanol molecules and excess lipid molecules into the growing pocket. Ultimately, the bilayer zips along the entire interface forming a liposome and pinching off the 1-octanol pocket in the form of a separate droplet. The continuous flow of the OA stream transporting the vesicles downstream provides some additional shear stress for the liposome-droplet separation to occur. It should be noted that after separation, the liposomes and 1-octanol droplets coexist in the device. If preferred, they may be separated downstream using inherent differences between the two, such as their dielectric constants, densities, fluorescence intensities or deformability. Attempts to separate the 1-octanol droplets from the liposomes are now being carried out in our lab. To our advantage, the octanol molecules do not dissolve in the OA phase, presumably since the excess lipids form a monolayer at the droplet–water interface. Similar budding-off of oil droplets from liposomes was reported before, albeit without control since the double-emulsion droplets needed to stick to a surface through nonspecific interactions^18^. Thus, instead of relying on the time-consuming solvent-extraction process, we realized a much quicker process (∼1,000 times faster) of physically separating the unwanted LO phase from the liposome. We coin this process ‘octanol-assisted liposome assembly (OLA)', in which the LO phase, along with excess lipids, gets completely separated from the liposome. OLA also eliminates the need to form ultrathin-shelled double-emulsion droplets in a critically fine-tuned process, instead offering a robust production technique. Note that we also tried using higher alcohols (1-nonanol and 1-decanol) as the LO phase. However, in those cases, the alcohol pockets did not separate from the double-emulsion droplets (Supplementary Table 1).

Figure 3a highlights the pinching-off process occurring at the junction at high temporal resolution. It can be seen that the LO phase (1-octanol) constantly surrounds the IA phase during the bubble formation, keeping it isolated from the OA phase across all steps in the process (Supplementary Movie 1). Small satellite 1-octanol droplets (∼1 μm) were generated occasionally (Fig. 2b and Supplementary Movie 1). We checked the encapsulation efficiency in our system using a fluorescent dye (Alexa Fluor 350, 0.5 mM) that was dissolved exclusively in the IA. As can be seen in Fig. 3b, the fluorescent IA phase got completely engulfed inside the double-emulsion droplet, with no visible leakage during pinching off, thus verifying an excellent encapsulation yield (Supplementary Movie 4). It should be noted that the LO phase volume forming the double-emulsion droplet can vary considerably, depending on the LO phase velocity. For example, while in Fig. 2b the dispensed LO phase volume was substantial and formed a prominent side pocket, the volume of the dispensed LO phase was smaller in case of Fig. 3a, and the pocket developed afterwards, not within the displayed time range.

Although we did not observe any visible pockets of remaining solvent, we cannot eliminate the possibility that some 1-octanol traces might remain in the bilayer. Beneficially, however, due to the low water miscibility of 1-octanol (0.54 gl^−1^ (ref. 30)), any trace 1-octanol molecules present in the bilayer will dissolve in the aqueous phase over time. Moreover, 1-octanol is a particularly favourable biocompatible organic solvent compared with other commonly used solvents such as *n*-decane, toluene and chloroform. For example, only a 2.5% loss in the enzymatic activity of epoxide hydrolase in yeast cells was reported in the presence of 10 v% 1-octanol^31^. Thus, the very low concentration of 1-octanol (0.05 v%, corresponding to 1-octanol's water miscibility) that can also get dissolved in the vesicle lumen will be highly unlikely to affect the function of encapsulated biomolecules.

### OLA-based liposomes are unilamellar

The unilamellarity of liposomes is a crucial parameter for biocompatible applications such as studying the function of membrane proteins and membrane transport through protein channels. To check whether the liposome boundary was actually composed of a single lipid bilayer, we inserted the bilayer-spanning protein pore, α-haemolysin. Monomers of this protein self-assemble as heptameric mushroom-shaped 1.5-nm wide pores in the phospholipid bilayers, which makes the bilayer permeable to small molecules (<2 kDa)^32^,^33^. We encapsulated α-haemolysin (2 μM) inside the liposomes along with a fluorescent dye Alexa Fluor 350 (0.5 mM), and subsequently observed the leakage of Alexa Fluor 350 into the OA phase (Fig. 4a and Supplementary Movie 5). Once formed, the vesicles indeed lost their internal fluorescence completely over a period of ∼2.5 min. Note that the leakage started even before the separation of 1-octanol droplet from the liposome, highlighting the rapid bilayer formation and exclusion of 1-octanol molecules into the pocket. A negative control without α-haemolysin showed only a minor reduction in the fluorescence due to photobleaching (Fig. 4b and Supplementary Movie 6). For quantification, the relative fluorescence intensity inside the vesicles was plotted as a function of time (Fig. 4c; Methods). The mean fluorescence of the α-haemolysin-containing vesicles exponentially decayed to zero with a decay constant of *k*≈0.4 s^−1^ (red circles in Fig. 4c, solid black line represents the fit). The mean fluorescence of the control vesicles showed only a marginal decay (blue squares). To test how much of this decay was due to the photobleaching of the fluorescent dyes, a bleaching assay was performed by flooding the post-junction channel with the IA phase and measuring the fluorescence intensity over time (magenta line). The obtained decay corresponded very well with that of the negative control, further verifying the non-leaky nature of the vesicles without α-haemolysin. We conclude that liposomes formed using OLA are indeed unilamellar and do not exhibit any significant leakage.

### Monodisperse liposomes with biologically relevant sizes

Another important parameter regarding liposome production methods is the size range and monodispersity of the liposomes. Although current microfluidic methods can easily achieve giant liposomes (> 50 μm), the lower size limit seems to be about 20 μm (refs 18, 20, 23; or rarely as small as 10 μm (ref. 26)). Using OLA, we decided to probe the lower size limit. We find that we can efficiently form liposomes as small as 5 μm. Figure 5c shows some representative liposomes of various sizes confined within the microfluidic channels. Figure 5a shows frequency histograms of four batches of liposomes formed in the size range of 5–20 μm. Solid curves are Gaussian fits to the individual histograms, while the arrows point out the mean diameters along with the corresponding s.d.'s (from left to right: 5.6±0.6, 10.8±0.5, 15.9±0.7 and 19.5±0.7 μm, see Methods). In each case, we got a narrow, monodisperse size distribution with the coefficient of variation ranging between 4 and 11% of the mean. The flow velocities of the IA and OA streams primarily govern the liposome diameter. The flow velocity of the IA stream governs how much fluid gets encapsulated within the liposome while the OA flow velocity determines how frequently the pinching off takes place. We obtained a linear relationship between the ratio of the two flow velocities (OA phase:IA phase) and the size of the resulting liposomes (Fig. 5b; Methods). The IA channel dimensions were also found to determine the minimum fluid volume that was encapsulated, setting a lower limit on the liposome size. For example, liposomes lower than 10 μm in diameter could not be obtained when the IA channel had dimensions of 10 × 11 μm (width × height). To obtain smaller liposomes, we reduced those dimensions to 3 × 5 μm and obtained liposomes with diameter of 5.6±0.6 μm. We expect that reducing the channel dimensions further will result in even smaller (∼1 μm) liposomes. Note that producing small liposomes in a controlled manner is highly important when working with protein systems that naturally function at this range of cell sizes and membrane curvature.

### Divisome proteins co-localize at the liposomal inner leaflet

Biocompatibility is a vital aspect of any liposome-forming technique. For many applications, one needs to encapsulate functional biomolecules inside the liposomes that often also have to attain interactions with the lipid membrane. We tested the biocompatibility of OLA-based liposomes by encapsulating two key bacterial divisome proteins, viz., FtsZ (labelled with Alexa Fluor 488) and sZipA (labelled with Alexa Fluor 647), inside the liposomes. FtsZ is a GTPase that polymerizes into filaments and is a key protein forming a proto-ring that directs the assembly of a division ring, ultimately resulting in cell division^34^. ZipA, which has a N-terminal hydrophobic domain that gets inserted into the membrane, acts as an anchoring point for FtsZ (ref. 35). We used sZipA, a soluble variant of ZipA that lacks the N-terminal region but is instead connected to a His-tag peptide. The His-tag interacts with lipids containing nickel in the headgroup, such as 1,2-dioleoyl-sn-glycero-3-[(N-(5-amino-1-carboxypentyl)iminodiacetic acid)succinyl] (nickel salt) (DGS-NTA(Ni)), thus rendering the function of sZipA identical to that of native ZipA (ref. 12). In the presence of DGS-NTA(Ni) lipids (90 mol% DOPC (1,2-dioleoyl-*sn*-glycero-3-phosphocholine)+10 mol% DGS-NTA(Ni)) and 2 mM GTP (to induce FtsZ polymerization), we observed a co-localization of FtsZ filaments and ZipA at the liposome membrane resulting in arc-like structures (Fig. 6a). When DGS-NTA(Ni) lipids were absent from the bilayer, FtsZ filaments and bundles were randomly distributed inside the liposomes (Fig. 6b). These experiments clearly show that OLA-based liposomes not only can efficiently encapsulate active proteins but also present a functional lipid bilayer suitable for interactions with biomolecules. It should be noted that the IA and OA phases contained buffers with physiological salt concentrations (150 mM KCl and 5 mM MgCl_2_; see Methods).

OLA is found to be robust to variations in the lipid composition, which is particularly important if one desires to mimic a certain membrane composition or wishes to tether or insert specific proteins at the membrane. DOPC was used as the lipid source for the initial development of OLA (Figs 1, 2b and 4). Later on, a mixture of DOPC and negatively charged 1,2-dioleoyl-*sn*-glycero-3-[phospho-*rac*-(3-lysyl(1-glycerol))] (DOPG) was used, which also worked equally well (Figs 2c and 3). Note that this particular lipid composition was used to mimic the charge density of the *Escherichia coli* polar lipid fraction (66.6 mol% DOPC+33.3 mol% DOPG)^36^. Lastly, the mixture of DOPC and DGS-NTA(Ni) also worked very well (Fig. 6).

## Discussion

**Figure 1:**
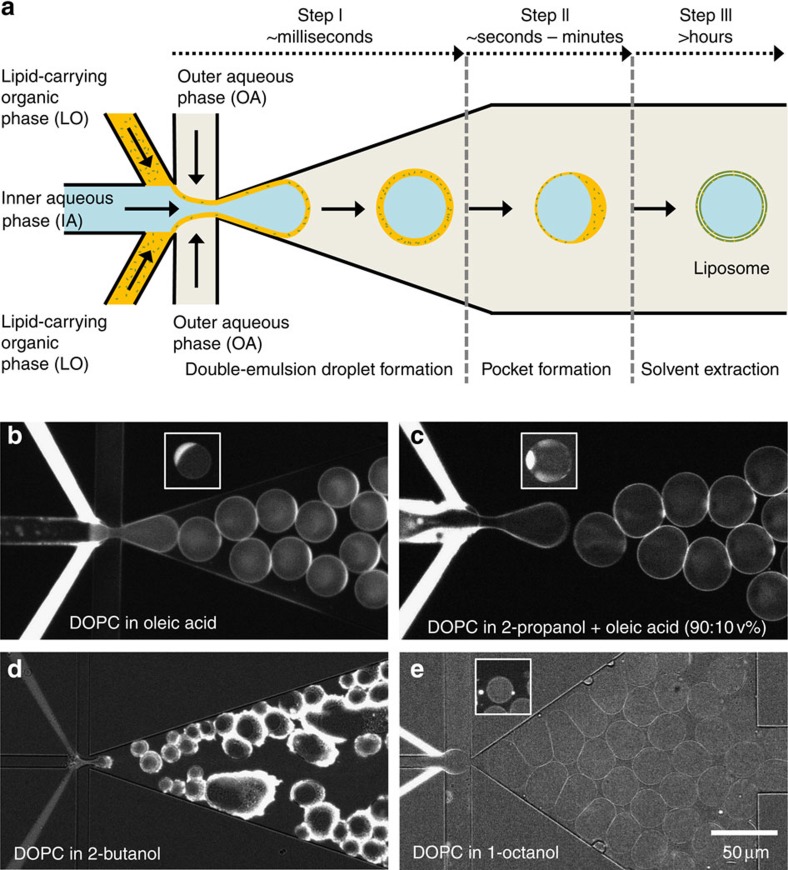
Double-emulsion droplet formation using different lipid-carrying organic phases. (**a**) Scheme representing the production of liposomes using a double-emulsion solvent-extraction mechanism in a microfluidic device. The formation of double-emulsion droplets and the subsequent formation of a residual oil pocket within the bilayer is relatively quick, but the solvent-extraction process is much slower (>10 h typically). Fluorescence images of attempted double-emulsion droplet formations using (**b**) oleic acid, (**c**) 90 v% 2-propanol+10 v% oleic acid, (**d**) 2-butanol and (**e**) 1-octanol as the LO phase. Corresponding images of the intermediate state of a liposome with an attached side pocket are shown in the insets. See Methods for solution compositions.

We present a simple and elegant microfluidic technique to form unilamellar, monodisperse and cell-sized liposomes. Named OLA, this double-emulsion droplet-based method uses 1-octanol, a biocompatible organic solvent as the lipid-carrying phase, which leads to a quick and clean physical solvent-extraction process. Our method circumvents the common problems such as remnants of organic solvents in the lipid bilayer and the time-consuming solvent-extraction associated with existing methods^17^. OLA initially forms a double-emulsion droplet, which immediately phase transforms into an intermediate state of an aqueous volume encircled by a lipid bilayer with a side-attached solvent pocket. Within a few minutes, the bilayer completely zips along the entire interface and the 1-octanol pocket spontaneously separates from the assembled liposome. Apart from the geometry of our microfluidic device (the six-way junction) that produces double-emulsion droplets in a single step, using 1-octanol as the LO phase makes OLA highly efficient and user-friendly over existing techniques. Generally, the potential to form double-emulsion droplets increases as the water miscibility of the solvent decreases, while at the same time, its biocompatibility decreases. 1-octanol achieves a fine balance between the two parameters as compared with other LO phases (Supplementary Table 1). For example, it was shown that using a better water-miscible compound like ethyl acetate (solubility: 64–80 gl^−1^ (ref. 37)) produces multilamellar thick-shelled lipid particles^38^. This result is similar to the one we obtained using 2-butanol as the LO phase (solubility: 181 gl^−1^ (ref. 39); Fig. 1d). We show the unilamellarity and biocompatibility of the OLA-based liposomes by inserting functional α-haemolysin pores in the membranes and by the localization of the encapsulated bacterial divisome proteins at the inner leaflet. OLA can produce liposomes with rates up to 75 Hz and with as small as 4% variation in the size, which is similar to other liposome-producing techniques^20^,^21^,^22^,^23^,^26^.

In addition, OLA offers several other important advantages. Since the liposomes can have varying membrane composition and can encapsulate high salt-containing biological buffers, they can be used as nanoreactors, that is, as functional protein-expression systems. Owing to the unilamellarity and use of a biofriendly organic solvent, OLA is a worthy platform to study physiochemical properties of biological membranes along with reconstituting membrane proteins. It is also perfectly suitable for the emerging field of bottom-up synthetic biology, which aims to construct cell-like assemblies using liposomes as the basic architectural scaffold. The main reasons that OLA-based liposomes fit so well for this purpose are the excellent encapsulation yield, which is important when trying to simultaneously encapsulate a large variety of biomolecules in defined ratios, an appropriate liposome size range similar to living cells (5–20 μm), and the possibility to immediately observe and manipulate the liposomes right after their formation. Finally, OLA-based liposomes could also find many applications in pharmaceutical sciences, for example, as potential drug and gene delivery systems.

## Methods

### LO phase preparation

Lipids (DOPC, DOPG, DGS-NTA(Ni) and Liss Rhod PE (1,2-dioleoyl-*sn*-glycero-3-phosphoethanolamine-N-(lissamine rhodamine B sulfonyl) (ammonium salt))) were purchased as solutions in chloroform from Avanti Polar Lipids, Inc. Chloroform was evaporated by passing a gentle stream of argon, and the lipids were further dried by desiccating for at least 2 h. A stock concentration (50–100 mg ml^−1^) was prepared by dissolving the lipids in ethanol and stored at −20 °C under argon atmosphere. The fluorescent lipid Liss Rhod PE was added for visualization (99.9 mol% DOPC+0.1 mol% Liss Rhod PE; 66.6 mol% DOPC+33.3 mol% DOPG+0.1 mol% Liss Rhod PE; or 89.9 mol% DOPC+10 mol% DGS-NTA(Ni)+0.1 mol% Liss Rhod PE). During experiments, the stock solution was dissolved in 1-octanol (Sigma-Aldrich Co.) to a final concentration of 2 mg ml^−1^ (unless specified otherwise).

### Soft lithography

Patterns were fabricated in silicon using e-beam lithography and dry etching as follows. A 4′′ diameter silicon wafer was cleaned in fuming nitric acid (100% HNO_3_) for 10 min in an ultrasonication bath, rinsed in deionized water and spin-dried. The wafer surface was then primed with hexamethyldisilazane (BASF SE) to enhance resist adhesion by spin-coating (1,000 r.p.m. for 1 min) and baking at 200 °C for 2 min. Next, negative resist NEB22A (Sumitomo Chemical Co., Ltd) was spin-coated (1,000 r.p.m. for 1 min) and the wafer was pre-baked at 110 °C for 3 min. A Leica EBPG 5000+ (acceleration voltage 100 kV and aperture 400 μm) was used to write the desired pattern on the coated wafer with a dose of 16 μC cm^−2^. The wafer was immediately post-baked at 105 °C for 3 min. MF322 (The Dow Chemical Company) was used for developing the patterns (30 s), followed by rinsing the wafer in diluted MF322 solution (10 v% MF322+90 v% water) for 15 s and finally in deionized water for 15 s.

Dry etching was done using Bosch deep reactive-ion etching^40^, with an inductive coupled plasma (ICP) reactive-ion etcher (Adixen AMS 100 I-speeder). The process consisted of alternate etching (sulphur hexafluoride, SF_6_) and passivation (octafluorocyclobutane, C_4_F_8_) cycles. The pressure was kept around 0.04 mbar. The etching step involved 200 s.c.c.m. SF_6_ for 7 s with the ICP power set to 2,000 W, while the capacitive coupled plasma (CCP) power (biased power) was switched off. The passivation step was 80 s.c.c.m. C_4_F_8_ for 3 s with the ICP power set to 2,000 W and the CCP power in chopped low frequency bias mode: 80 W, ON 10 ms, OFF 90 ms. Constant temperature (10 °C) was maintained during the entire process. Temperature of the main chamber was kept at 200 °C. The sample holder was held at 200 mm from the source. The etching rate was∼3 μm min^−1^. The heights were measured using a stylus profiler, DektakXT (Bruker Corporation). Finally, the wafer was again cleaned in 100% HNO_3_ for 5 min in an ultrasonication bath, rinsed in deionized water and spin-dried. The wafer was then rendered hydrophobic by exposing it to (tridecafluoro-1,1,2,2-tetrahydrooctyl) trichlorosilane (ABCR GmbH & Co.) in partial vacuum for at least 12 h.

Microfluidic devices were made by pouring PDMS (Sylgard 184, Dow Corning GmbH), at a mass ratio 15:1, on the wafer and baking at 80 °C for 4 h. The PDMS block was then peeled off from the wafer and holes were punched into it using a biopsy punch (World Precision Instruments, inner diameter 750 μm). The PDMS was then cleaned with isopropanol and dried with nitrogen. Glass slides were also coated with a thin PDMS layer as follows. A thin layer of PDMS (mass ratio of 15:1) was applied to a clean hydrophobic silicon wafer. Clean glass slides were lightly pressed on the wafer until they were completely immersed in PDMS and baked at 80 °C for 4 h. The cured PDMS layer was peeled off and the glass slides were carefully removed, with their underside bearing a thin PDMS layer. The PDMS block and the coated glass slide were then exposed to oxygen plasma for ∼10 s using a Plasma-Preen system (Plasmatic Systems, Inc.). Immediately after the plasma treatment, the glass slide was bonded to the PDMS block. The device was further baked at 80 °C for ∼20 min. Microfluidic flow control system (Fluigent GmbH) along with the MAESFLO software (version 3.2.1) were used to flow the solutions into the microfluidic device using appropriate connectors and tubings (Tygon Microbore Tubing).

For producing liposomes in the size range 10–20 μm, the microstructures were ∼11 μm in height, and the widths of the microchannels at the junction were as follows: IA channel, 10 μm; LO channel, 5.4 μm; OA channel, 20 μm; and post-junction channel, 200 μm.

For producing 5-μm diameter liposomes, the microstructures were ∼5 μm in height and the widths of the microchannels at the junction were as follows: IA channel, 3 μm; LO channel, 6.6 μm; OA channel, 10 μm; post-junction channel, 100 μm.

### Surface treatment

Channels downstream of the junction were rendered hydrophilic by adsorbing PVA polymers to the PDMS surface. PVA solution (50 mg ml^−1^, 87–90% hydrolysed, molecular weight 30,000–70,000, Sigma-Aldrich Co.) was injected from the OA channels and was prevented from entering the IA and the LO channels by applying positive air pressure on them (Supplementary Fig. 1). After a short incubation time, to allow PVA polymers assemble on the surface (∼5 min), vacuum was applied at the outlet and the PVA solution was removed from the device. The device was then baked at 120 °C for ∼15 min to heat immobilize the PVA polymers onto the surface. Successfully PVA-treated devices were used to produce liposomes up to 4 h. It should be noted that this does not correspond to the maximum time limit over which a device can be used, but to the duration of the experiments.

### Image acquisition and processing

An Olympus IX81 inverted microscope equipped with epifluorescence illumination, appropriate filter sets, × 20 UPlanSApo (numerical aperture 0.75) and × 60 PlanApo (numerical aperture 1.45, oil) objectives (Olympus) was used to perform the experiments. The images were recorded using Neo sCMOS camera (Andor Technolgy Ltd.) and micromanager software (version 1.4.14)^41^. Image processing was performed using ImageJ and MATLAB through self-written scripts.

### Unilamellarity check using α-haemolysin protein pores

For both α-haemolysin-containing and not-containing liposomes, their mean fluorescence intensities, depending on the amount of Alexa Fluor 350 present in the lumen, were calculated after appropriate background subtraction. For each time point, data points within one s.d. were used for the analyses. This was done to avoid spurious errors arising from less-bright liposomes with functional pores inserted into the membrane even before the start of the data acquisition (in case of α-haemolysin-containing liposomes) and a small fraction of defective leaky liposomes (in case of control liposomes). Each time point corresponds to a population average of all the liposomes within the field of view. Even though the fluid flow was kept to a minimum, the field of view for each time point might contain different liposomes.

### Size dependence on OA/IA phase velocity

Each of the analysed liposome populations was obtained from an individual experiment, where at least 150 vesicles were analysed for each data point, as described below. Fluorescence images of liposomes encapsulated with Alexa Fluor 350 were properly thresholded and binarized. The cross-sectional area *A* of each of the liposomes was calculated. Knowing the microchannel height *h*, the cross-sectional area *A*_max_ of the largest liposome that would fit without the liposome getting squeezed was calculated as *πh*^2^/4. When *A*≤*A*_max_, the corresponding diameter was calculated as *d*=2(*A*/*π*)^0.5^. When *A*⩾*A*_max_, the liposome was squeezed and would have an ellipsoid form. The corresponding diameter was then calculated as *d*=2(*A*·*h*/2*π*)^0.33^.

Flow velocities of IA and OA streams were calculated separately for each stream using the relation *v*=*Q*/*a*, where *v* is the flow velocity, *Q* is flow rate and *a* is the cross-sectional area of that particular channel. *Q* was calculated as Δ*p*·*w*·*h*^3^(1−0.63 *h*/*w*)/12·*η*·*L*, where Δ*p* is pressure difference across the channel, *η* is the fluid viscosity and *L*, *w* and *h*, respectively, are the channel length, width and height^42^. Viscosities of different solutions were measured using Dv2T viscometer (Brookfield Engineering Laboratories, Inc., USA).

### Solution compositions

**Figure 2:**
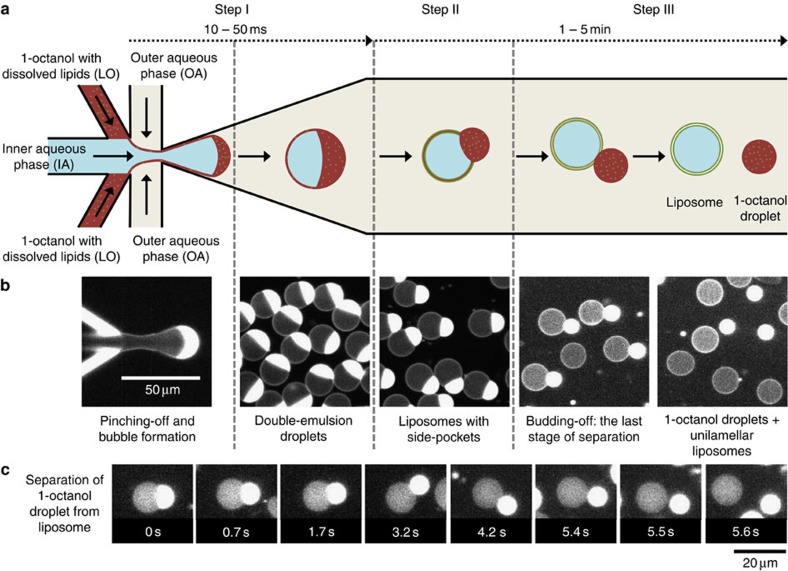
Octanol-assisted liposome assembly. (**a**) Schematic representation showing the working principle of on-chip production of liposomes using OLA. Step I: the IA phase and the surrounding LO phase are hydrodynamically focused and subsequently pinched off by the two OA streams to form a double-emulsion droplet. Step II: a lipid bilayer assembles along the interface while 1-octanol molecules, along with excess lipids, spontaneously phase separate to form a prominent pocket. Step III: the 1-octanol pocket containing excess lipids spontaneously separates in the form of a droplet to form a fully assembled unilamellar liposome. (**b**) Corresponding fluorescence images showing each of the steps described above. (**c**) Temporal-resolution sequences showing the separation of the 1-octanol droplet from the liposome. The first frame of the sequence in **c** was obtained about 1 min after the double-emulsion droplet formed. See Methods for solution compositions.

**Figure 3:**
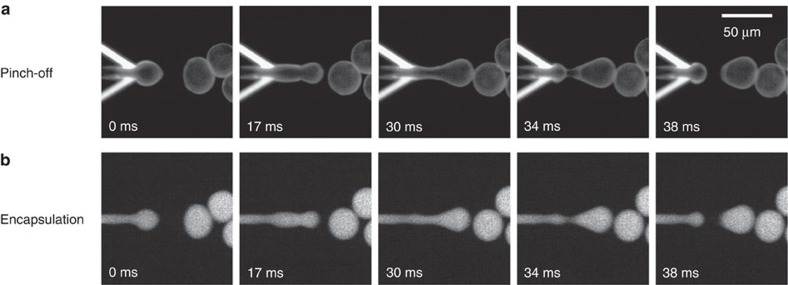
Pinching-off process and the concurrent encapsulation. High-temporal-resolution sequences showing (**a**) the pinching-off process to form a double-emulsion droplet; (**b**) corresponding images showing the encapsulation of the fluorescent IA phase (Alexa Fluor 350) during the pinching-off process. See Methods for solution compositions.

The different compositions of IA, LO and OA phases used in the experiments are given below. To prevent the coalescence of liposomes and to increase their stability, a pluronic surfactant (poloxamer 188, 50 mg ml^−1^) was added to the IA and OA phases. The surfactant was not added to the IA phase while encapsulating bacterial divisome proteins, to retain the protein functionality. The OA phase consisted 15 v% glycerol to improve the pinching-off process and to stabilize the liposomes. In the initial experiments 15 v% ethanol was added to the OA phase to extract 1-octanol (Figs 2c and 3), but this was found unnecessary and the method worked equally well without it.

**Figure 4:**
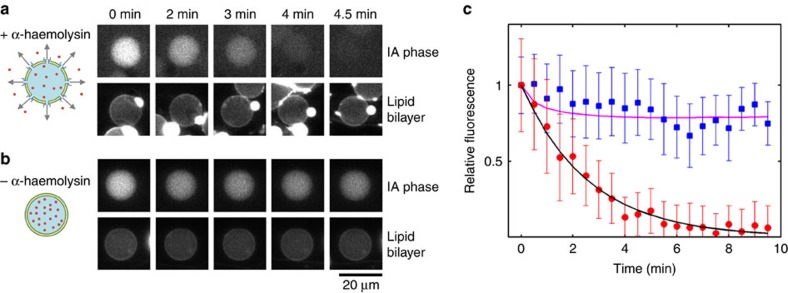
Unilamellarity of the liposomes. (**a**) Addition of the membrane protein pore α-haemolysin (2 μM) to the IA phase leads to leaky liposomes, which can be seen from the decreasing fluorescence of Alexa Fluor 350 over time. Note that the leakage already starts even before the separation of 1-octanol droplet from the liposome. (**b**) A negative control in the absence of α-haemolysin shows only a marginal decay in the fluorescence. (**c**) Relative fluorescence observed inside the vesicles against time (red circles, with α-haemolysin; blue squares, without α-haemolysin; *n*⩾26 at each time point). Error bars indicate corresponding s.d.'s. Solid black line represents an exponential decay (*k*≈0.4 s^−1^). The magenta line represents the bleaching control. See Methods for solution compositions.

**Figure 6:**
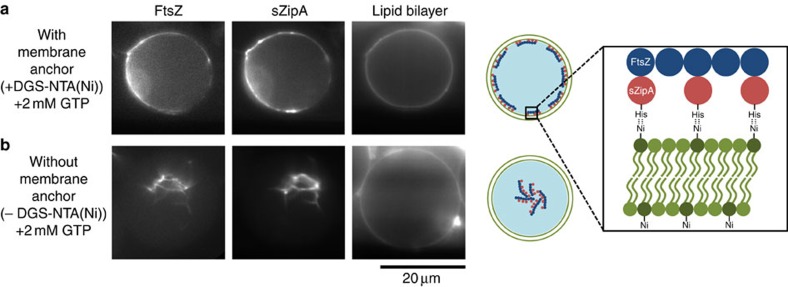
Localization of bacterial divisome proteins at the inner leaflet of the liposome membrane. (**a**) sZipA with a His-tag binds to the DGS-NTA(Ni) lipids present in the membrane and in turn recruits the FtsZ filaments, forming arc-like structures localized at the membrane. (**b**) In the absence of DGS-NTA(Ni) lipids, no such recruitment to the membrane is seen and FtsZ filaments and bundles cluster together within the vesicle lumen, co-localized with sZipA. In both the experiments, 2 mM GTP was added to the IA phase to induce FtsZ polymerization. See Methods for solution compositions.

IA phase (all solutions were prepared in Milli-Q water): 50 mg ml^−1^ poloxamer 188 (Figs 1 and 2b); 0.5 mM Alexa 350 and 50 mg ml poloxamer 188 (Figs 2c and 3); 0.5 mM Alexa 350, 10 mM HEPES (pH 7.5), 15 v% glycerol and 50 mg ml^−1^ poloxamer 188, with or without 2 μM α-haemolysin (Fig. 4); 12 μM FtsZ, 6 μM ZipA, 2 mM GTP, 150 mM KCl, 5 mM MgCl_2_, 50 mM TrisHCl (pH 7.5), 15 v% glycerol and 10 mg ml^−1^ PVA (Fig. 6).

LO phase: 5 mg ml^−1^ lipids in oleic acid (Fig. 1b), 2 mg ml^−1^ lipids in 90 v% 2-propanol+10 v% oleic acid (Fig. 1c), 2 mg ml^−1^ lipids in 2-butanol (Fig. 1d) and 1 mg ml^−1^ lipids in 1-octanol (Fig. 1e; lipid composition: 99.9 mol% DOPC+0.1 mol% Liss Rhod PE); 2 mg ml^−1^ lipids (99.9 mol% DOPC+0.1 mol% Liss Rhod PE) in 1-octanol (Figs 2b and 4); 2 mg ml^−1^ lipids (66.6 mol% DOPC+33.3 mol% DOPG+0.1 mol% Liss Rhod PE) in 1-octanol (Figs 2c and 3); and 2 mg ml^−1^ lipids (89.9 mol% DOPC+10 mol% DGS-NTA(Ni)+0.1 mol% Liss Rhod PE) in 1-octanol (Fig. 6).

OA phase (all solutions were prepared in Milli-Q water): 50 mg ml^−1^ poloxamer 188, 15 v% ethanol and 15 v% glycerol (Figs 1, 2b and 3); 50 mg ml^−1^ poloxamer 188 and 15 v% glycerol (Figs 2c and 4); 50 mg ml^−1^ poloxamer 188, 150 mM KCl, 5 mM MgCl_2_, 50 mM TrisHCl (pH 7.5), 15 v% glycerol and 10 mg ml^−1^ PVA (Fig. 6).

FtsZ and sZipA proteins were a generous gift from Germán Rivas; purification and labelling is described in ref. 12. Composition of the protein stock solutions was as follows: 420 μM FtsZ in 50 mM TrisHCl (pH 7.5), 500 mM KCl, 5 mM MgCl_2_, EDTA 0.1 mM and 10 v% of glycerol; 47 μM FtsZ-Alexa Fluor 488 in 50 mM TrisHCl (pH 7.5), 500 mM KCl, 5 mM MgCl_2_ and 10 v% of glycerol; 30 μM sZipA in 50 mM TrisHCl (pH 7.5), 500 mM KCl, 5 mM MgCl_2_ and 5 v% of glycerol; 7 μM sZipA-Alexa Fluor 647 in 50 mM TrisHCl (pH 7.5), 500 mM KCl, 5 mM MgCl_2_ and 5 v% of glycerol. The stock solutions were stored at −20 °C. For the experiments, the labelled and the unlabelled proteins were mixed in the molar ratio of 1:9.

## Additional information

**How to cite this article:** Deshpande, S. *et al*. Octanol-assisted liposome assembly on chip. *Nat. Commun.* 7:10447 doi: 10.1038/ncomms10447 (2016).

## Supplementary Material

Supplementary Information Supplementary Figure 1, Supplementary Table 1 and Supplementary References

Supplementary Movie 1 Double-emulsion droplet formation. IA phase: 0.5 mM Alexa Fluor 350, 50 mg/mL poloxamer 188; LO phase: 2 mg/mL lipids (99.9 mol% DOPC + 0.1 mol% Liss Rhod PE) in 1-octanol; OA phase: 50 mg/mL poloxamer 188, 15 v% ethanol, 15 v% glycerol.

Supplementary Movie 2 Pocket formation. IA phase: 0.5 mM Alexa Fluor 350, 50 mg/mL poloxamer 188; LO phase: 2 mg/mL lipids (99.9 mol% DOPC + 0.1 mol% Liss Rhod PE) in 1-octanol; OA phase: 50 mg/mL poloxamer 188, 15 v% ethanol, 15 v% glycerol.

Supplementary Movie 3 Separation of the 1-octanol droplet from the liposome. IA phase: 0.5 mM Alexa Fluor 350, 50 mg/mL poloxamer 188; LO phase: 2 mg/mL lipids (66.6 mol% DOPC + 33.3 mol% DOPG) in 1-octanol; OA phase: 50 mg/mL poloxamer 188, 15 v% glycerol.

Supplementary Movie 4 Encapsulation of inner aqueous phase. IA phase: 0.5 mM Alexa Fluor 350, 50 mg/mL poloxamer 188; LO phase: 2 mg/mL lipids (99.9 mol% DOPC + 0.1 mol% Liss Rhod PE) in 1-octanol; OA phase: 50 mg/mL poloxamer 188, 15 v% ethanol, 15 v% glycerol.

Supplementary Movie 5 Leakage of the IA phase through a-hemolysin pores inserted into the lipid bilayer. IA phase: 2 μM a-hemolysin, 0.5 mM Alexa350, 50 mg/mL poloxamer 188, 15 v% glycerol, 10 mM HEPES (pH 7.5); LO phase: 2 mg/mL lipids (99.9 mol% DOPC + 0.1 mol% Liss Rhod PE) in 1-octanol; OA phase: 50 mg/mL poloxamer 188, 15 v% glycerol.

Supplementary Movie 6 No leakage of the IA phase in the absence of a-hemolysin pores. IA phase: 0.5 mM Alexa350, 50 mg/mL poloxamer 188, 15 v% glycerol, 10 mM HEPES (pH 7.5); LO phase: 2 mg/mL lipids (99.9 mol% DOPC + 0.1 mol% Liss Rhod PE) in 1-octanol; OA phase: 50 mg/mL poloxamer 188, 15 v% glycerol.

## Figures and Tables

**Figure 5:**
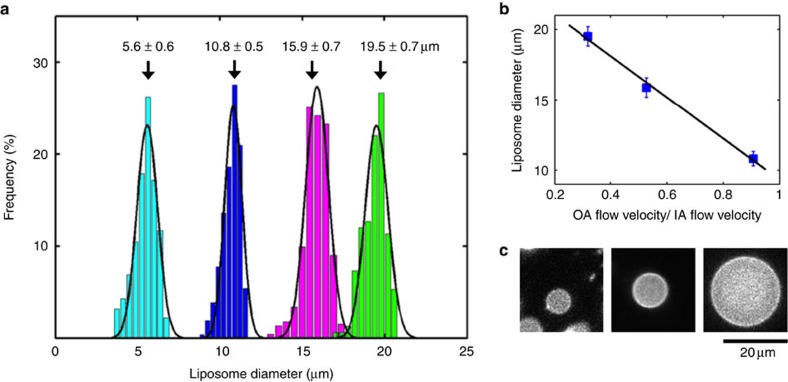
Size range and monodispersity of liposomes. (**a**) Frequency histograms showing the biologically relevant size range of OLA-based liposomes (5–20 μm) and a high monodispersity (4–11%) for each of the populations (*n*⩾150 for each population). Solid lines are Gaussian fits for individual histograms while arrows point out the means diameters and the corresponding s.d.'s (5.6±0.6, 10.8±0.5, 15.9±0.7 and 19.5±0.7 μm, see Methods). Note that each population was obtained under different experimental conditions, that is, in each case the distribution is unimodal. (**b**) Liposome diameter versus OA flow velocity:IA flow velocity. Error bars indicate corresponding s.d.'s. The line indicates a linear fit. (**c**) Representative fluorescence images showing liposomes of various diameters.
