# Supplementary material for: Octanol-assisted liposome assembly on chip
Source: Nat Commun. 2016 Jan 22;7:10447. doi: 10.1038/ncomms10447 (PMC4735860; doi:10.1038/ncomms10447)
Supplement: Supplementary Information — Supplementary Figure 1, Supplementary Table 1 and Supplementary References [file ncomms10447-s1.pdf]

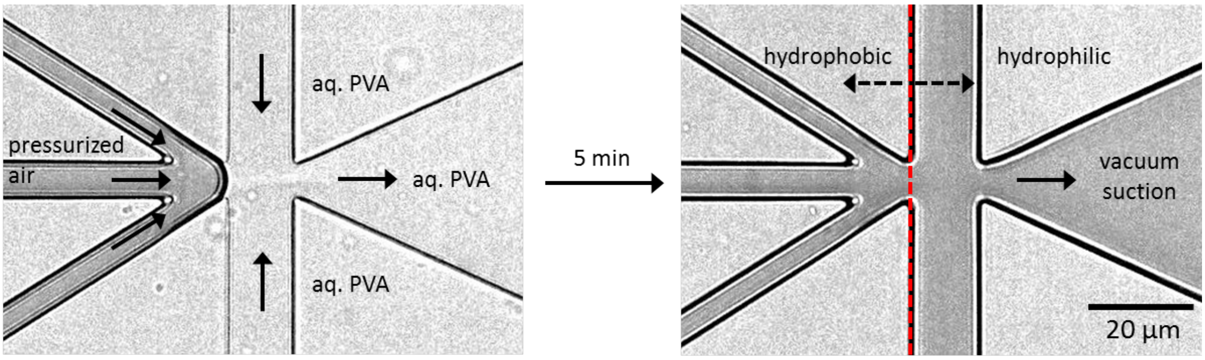

Supplementary Figure 1: Polyvinyl alcohol treatment of the microfluidic device to render it partially hydrophilic. Aqueous PVA solution (50 mg/mL) is injected through the two OA channels while a positive air pressure prevents it from entering the IA and LO channels. After an incubation time of ~ 5 minutes, the PVA solution is removed by applying vacuum at the outlet, resulting in a pre-junction hydrophobic part and a post-junction PVA-coated hydrophilic part.

Supplementary Table 1: Potential of different LO phases to form double-emulsion droplets.

LO phase	Solubility in water (gL^{-1})	Double-emulsion droplet formation
Ethanol/ 1-propanol/ 2-propanol	Miscible	Did not form double-emulsion droplets as the three phases simply mixed with one another.
10 v% oleic acid + 90 v% 2-propanol	Partially miscible	Stable double-emulsion droplets with smaller oil pockets could be obtained.
1-butanol	63-68[1]	Not possible
2-butanol	181[2]	Multilamellar thick-shelled double-emulsion droplets were obtained in an uncontrolled way.
Isobutanol	66.5-90.9[3]	Not possible
1-pentanol	22[4]	Lipids did not dissolve properly, leading to aggregation.
Isoamyl alcohol	26.7[5]	Lipids did not dissolve properly, leading to aggregation.
3-pentanol	55 (at 30°C)[6]	Lipids did not dissolve properly, leading to aggregation.
1-hexanol	5.9[7]	Unstable double-emulsion droplets formed sporadically.
1-heptanol	1.67[8]	Unstable double-emulsion droplets formed sporadically.
1-octanol	0.54[9]	Stable double-emulsion droplets could be formed, further leading to the separation of the 1-octanol pocket (OLA).
1-nonanol	0.14[10]	Unstable double-emulsion droplets were formed.
1-decanol	0.037[11]	Stable double-emulsion droplets could be formed but the pockets did not separate.
Oleic acid	Immiscible	Stable double-emulsion droplets could be formed but the pockets did not separate.

All the measurements are recorded at 25°C, unless specified otherwise.

Supplementary references

1. Hazardous Substances Data Bank. PubChem Compound Database; CID=263. at <https://pubchem.ncbi.nlm.nih.gov/compound/263#section=Solubility> (accessed December 11, 2015)

2. Hazardous Substances Data Bank. PubChem Compound Database; CID=6568. at <https://pubchem.ncbi.nlm.nih.gov/compound/6568#section=Solubility> (accessed December 11, 2015)

3. Hazardous Substances Data Bank. PubChem Compound Database; CID=6560. at <https://pubchem.ncbi.nlm.nih.gov/compound/6560#section=Solubility> (accessed December 11, 2015)

4. Hazardous Substances Data Bank. PubChem Compound Database; CID=6276. at <https://pubchem.ncbi.nlm.nih.gov/compound/6276#section=Solubility> (accessed December 11, 2015)

5. Hazardous Substances Data Bank. PubChem Compound Database; CID=31260. at <https://pubchem.ncbi.nlm.nih.gov/compound/31260#section=Solubility> (accessed December 11, 2015)

6. International Chemical Safety Cards. PubChem Compound Database; CID=11428. at <https://pubchem.ncbi.nlm.nih.gov/compound/11428#section=Solubility> (accessed December 11, 2015)

7. Hazardous Substances Data Bank. PubChem Compound Database; CID=8103. at <https://pubchem.ncbi.nlm.nih.gov/compound/8103#section=Solubility> (accessed December 11, 2015)

8. Hazardous Substances Data Bank. PubChem Compound Database; CID=8129. at <https://pubchem.ncbi.nlm.nih.gov/compound/8129#section=Solubility> (accessed December 11, 2015)

9. Hazardous Substances Data Bank. PubChem Compound Database; CID=957. at <https://pubchem.ncbi.nlm.nih.gov/compound/957#section=Solubility> (accessed December 11, 2015)

10. Hazardous Substances Data Bank. PubChem Compound Database; CID=8914. at <https://pubchem.ncbi.nlm.nih.gov/compound/8914#section=Solubility> (accessed December 11, 2015)

11. Hazardous Substances Data Bank. PubChem Compound Database; CID=8174. at <https://pubchem.ncbi.nlm.nih.gov/compound/8174#section=Solubility> (accessed December 11, 2015)
